# A Recap of Heme Metabolism towards Understanding Protoporphyrin IX Selectivity in Cancer Cells

**DOI:** 10.3390/ijms23147974

**Published:** 2022-07-19

**Authors:** Martin Kiening, Norbert Lange

**Affiliations:** Institute of Pharmaceutical Sciences of Western Switzerland, University of Geneva, Rue Michel-Servet 1, 1211 Geneva, Switzerland; martin.kiening@unige.ch

**Keywords:** aminolevulinic acid, protoporphyrin IX, cancer, photodynamic diagnosis, photochemotherapy, theranostics

## Abstract

Mitochondria are essential organelles of mammalian cells, often emphasized for their function in energy production, iron metabolism and apoptosis as well as heme synthesis. The heme is an iron-loaded porphyrin behaving as a prosthetic group by its interactions with a wide variety of proteins. These complexes are termed hemoproteins and are usually vital to the whole cell comportment, such as the proteins hemoglobin, myoglobin or cytochromes, but also enzymes such as catalase and peroxidases. The building block of porphyrins is the 5-aminolevulinic acid, whose exogenous administration is able to stimulate the entire heme biosynthesis route. In neoplastic cells, this methodology repeatedly demonstrated an accumulation of the ultimate heme precursor, the fluorescent protoporphyrin IX photosensitizer, rather than in healthy tissues. While manifold players have been proposed, numerous discrepancies between research studies still dispute the mechanisms underlying this selective phenomenon that yet requires intensive investigations. In particular, we wonder what are the respective involvements of enzymes and transporters in protoporphyrin IX accretion. Is this mainly due to a boost in protoporphyrin IX anabolism along with a drop of its catabolism, or are its transporters deregulated? Additionally, can we truly expect to find a universal model to explain this selectivity? In this report, we aim to provide our peers with an overview of the currently known mitochondrial heme metabolism and approaches that could explain, at least partly, the mechanism of protoporphyrin IX selectivity towards cancer cells.

## 1. Introduction

Since its discovery in 1987 by Malik and Lugaci [1], the selective accumulation of the photosensitizer protoporphyrin IX (PpIX) in neoplastic cells upon administration of 5-aminolevulinic acid (5-ALA) has been extensively reported [2,3,4,5]. However, the mechanisms underlying this crucial feature are still unclear. This is partially due to incomplete knowledge of the heme metabolism, to an obvious insufficient hindsight, but also because of recurrent discrepancies from one study to another. This review is an update of the current knowledge regarding the heme synthesis and degradation, and the high selectivity of PpIX production mechanism for neoplasms.

**Figure 1:**
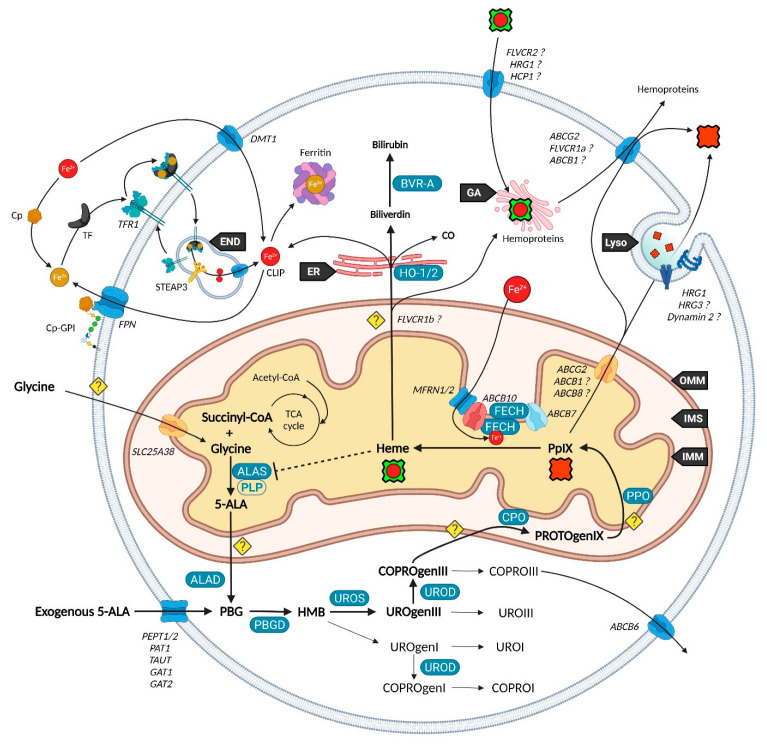
Current vision of the major players of heme metabolism and their interactions. Endoplasmic Reticulum (ER), Golgi Apparatus (GA), Outer/Inner Mitochondrial Membrane (OMM/IMM), Inner Mitochondrial Space (IMS), Endosome (END), and Lysosome (Lyso). Porphobilinogen (PBG), Hydroxymethylbilane (HMB), Uroporphyrinogen (UROgen), Coproporphyrinogen (COPROgen), and Protoporphyrinogen (PROTOgenIX). For other acronyms, please refer to the table at the end.

Unlike other molecules used in photodynamic therapy (PDT) and diagnosis (PDD), 5-ALA is the only naturally occurring agent. Considered present in all aerobic cells, it is a prime player in heme biosynthesis, whose penultimate by-product is PpIX. This pathway is highly regulated by eight enzymes, evenly distributed between mitochondria and the cytoplasm (Figure 1). Although the occurrence of a change in their expression can lead to severe disorders termed porphyria, often associated with skin photosensitivity, the control of this cycle can turn it into a potent tool. Since the conversion of 5-ALA allows to differentiate a normal from a cancerous cell by fluorescence, numerous 5-ALA derivatives have been synthetized to offer an optimized way to perform fluorescence-guided surgery of a wide variety of tumors [6].

However, a thorough understanding of the whole pathway is still required as it could create new treatment approaches on the avenue of theranostics. More essentially, why is protoporphyrin IX accretion so selective toward neoplastic cells?

## 2. Heme Metabolism—A Complex Network Tightly Regulated by Enzymes, Transporters and Other Metabolites

### 2.1. Biosynthesis of Iron Protoporphyrin IX

Branches surrounding the pathway of 5-ALA conversion into heme have become more and more exhaustive since Shemin and Rittenberg discovered the initial requirement of glycine in 1946 [7].

Heme, protoheme or iron protoporphyrin IX are a prosthetic complex of ferrous iron and PpIX that comes into play in major processes such as electron transfer chains, respiratory complexes, oxygen transport and storage of metal ions.

While 5-ALA exogenous administration leads to protoheme synthesis, the building block of tetrapyrroles is naturally formed in the mitochondrial matrix through the so-called “Shemin pathway” or C-4 [8,9]. Indeed, succinyl-CoA previously obtained by the tricarboxylic acid (TCA) or Krebs cycle is condensed with glycine into 5-ALA thanks to the enzyme ALA-synthase (ALAS, E.C. 2.3.1.37) and its cofactor pyridoxal-5′-phosphate (PLP), before export to the cytosol [10]. This single stage explains why the PpIX-mediated retro-negative feedback that inhibits ALAS can be circumvented by a straight 5-ALA uptake. Then, a second enzyme, ALA-dehydratase (ALAD, E.C. 4.2.1.24) converts two cytosolic 5-amino-4-oxopentanoic acids into the pyrrole derivative porphobilinogen via an asymmetric condensation. The association of four porphobilinogen catalyzed by the porphobilinogen deaminase (PBGD, E.C. 2.5.1.61) subsequently leads to the formation of a linear tetrapyrrolic structure, the 1-hydroxymethylbilane or pre-uroporphyrinogen. Uroporphyrinogen III cosynthase (UROS, E.C. 4.2.1.75) works in tandem with PBGD to close the cycle and enable the formation of the uroporphyrinogen III tetrapyrrolic cycle. This very first cycle is actually composed of eight 5-ALA molecules, the only source of carbon and nitrogen all along the pathway. From this point, decarboxylations are monitored along two stages. Primarily, the four acetate groups are decarboxylated into methyls by uroporphyrinogen decarboxylase (UROD, E.C. 4.1.1.37), turning uroporphyrinogen III into coproporphyrinogen III. Secondly, coproporphyrinogen III oxidase (CPO, E.C. 1.3.3.3) that localizes to the intermembrane space converts two out of four propionate residues into vinyls. The generated protoporphyrinogen IX undergoes further oxidation at the inner mitochondrial membrane by the protoporphyrinogen oxidase (PPO, E.C. 1.3.3.4). The hydrophobic PpIX obtained at this stage is the ultimate heme precursor. Its light-related features of fluorescence and photosensitization finally disappear in heme, when ferrous iron is inserted inside the aromatic structure by ferrochelatase (FECH, E.C. 4.99.1.1) localized at the inner flank of the inner mitochondrial membrane [11].

The synthesis of additional products of the heme biosynthesis pathway was recently explained. Indeed, the hydroxymethylbilane intermediary can spontaneously cyclize and form uroporphyrinogen I, albeit the UROS reaction leading to uroporphyrinogen III overrides it [12]. Similar to its isomer, uroporphyrinogen I can either be oxidized into uroporphyrin I, potentially by various cytochrome P450 isoenzyme catalyzation, or decarboxylated by UROD into coproporphyrinogen I that can in turn undergo spontaneous oxidation into coproporphyrin I. On the main path, uroporphyrinogen III can be transformed to uroporphyrin III, and coproporphyrinogen III can be turned into its coproporphyrin III oxidized counterpart. Both hydrophilic uroporphyrins and coproporphyrins display fluorescence properties very close to PpIX.

### 2.2. Utilization and Degradation of Iron Protoporphyrin IX

Heme is a ubiquitous component of every eukaryotic cell that plays a crucial role in their survival and behavior. However, an excess of this hydrophobic molecule is toxic to cells. It leads to the direct generation of reactive oxygen species (ROS) that in turn induce lipid peroxidation, DNA damage as well as protein damage and aggregation. Consequently, heme must be degraded by cells to prevent such deleterious effects. This role is endorsed by the heme oxygenase system. Heme oxygenase 1 and 2 (HO-1 and HO-2, E.C. 1.14.14.18) are famous for their role in tetrapyrrolic cycle opening and Fe^2+^ extraction that produces carbon monoxide (CO) and biliverdin. Located to the endoplasmic reticulum, HO-1 is expressed in most tissues and considered as a protective enzyme due to its inducibility by a profusion of factors. In contrast, HO-2 that contains heme regulatory motifs (HRM) is not induced by such factors but under oxidative stress conditions. Biliverdin reductase-A (BVR-A, E.C. 1.3.1.24) localizes to the cytosol and subsequently transforms biliverdin into bilirubin, that both, as well as HO-1 and CO, display important antioxidant properties [13,14,15]. The fate of the highly lipophilic bilirubin in cells, other than its absorption by hepatocytes, is not well described. The glucuronidation reaction by UDP-glucuronosyltransferase 1A1 (UGT1A1, E.C. 2.4.1.17) is known to transform such lipophilic substrates into hydrophilic metabolites that can subsequently move from hepatocytes to the bile and be excreted [16]. UGT1A1 and other members of its family where recently confirmed to be expressed in keratinocytes, suggesting that a similar elimination mechanism takes place in other cell types [17]. Additionally, the whole balance between heme and bilirubin might be controlled by a double negative feedback inhibition from bilirubin that would inhibit BVR-A [18] and from biliverdin that would inhibit heme oxygenase [19].

### 2.3. Transporters of the Heme Metabolism: Where Are We Now?

The functioning of the heme biosynthesis pathway is far from our full understanding, as attested by the bunch of inconsistencies on the involvement and role of transporters. Nevertheless, several of them are now unanimously accepted as heme metabolism milestones.

It was recently discovered that, in order to produce 5-ALA, cells import glycine through the mitochondrial glycine transporter localized at the inner mitochondrial membrane (IMM) and encoded by the SLC25A38 solute carrier gene [20]. The newly synthetized 5-ALA then moves out of the mitochondrion by a still obscure way. Previous studies suggested that SLC25A38 facilitates the exchange of glycine and 5-ALA through the mitochondrial membrane [21] and that the ATP-binding cassette subfamily B member 10 (ABCB10) is essential for 5-ALA export to the cytoplasm [22]. In contrast, it was more recently shown that silencing of ABCB10 in zebrafish and murine Friend erythroleukemia cells did not reduce the 5-ALA cytoplasmic concentration and thus rejected a direct interaction of ABCB10 with 5-ALA to export the latter [23].

Concerning the exogenous 5-ALA administration, due to its high hydrophilicity, crossing the cell barrier toughly occurs through passive diffusion, as this is the case for some derivatives (e.g., containing an ester moiety). Active transport and facilitated diffusion are more likely, then many intermediates have been proposed for this uptake, notably based on the idea of a structural similarity between the δ-amino acid 5-ALA, GABA and β-amino acids β-alanine and Taurine. Mainly, PEPT1 (SLC15A1), PEPT2 (SLC15A2), PAT1 (SLC36A1) and GABA neurotransmitter transporters TAUT (Taurine transporter SLC6A6), GAT1 (SLC6A1) and GAT2 (SLC6A13) demonstrated the ability to transport 5-ALA [24,25,26,27,28,29,30].

Once in the cytoplasm, it has been pointed that the ABCB6 transporter inserted inside the outer mitochondrial membrane (OMM) facilitates the mitochondrial crossing by the sub-product coproporphyrinogen III and subsequently acts as a checkpoint that enhances or prevents further heme production [31,32]. Nonetheless, the mitochondrial localization of ABCB6 is controversial and more and more attributed to lysosomes as well as to the plasma membrane [33,34,35]. In fact, ABCB6 is thought to be a potent activatable tetrapyrrole transporter that enables the cells to remove the cytosolic coproporphyrin III excess [31,36] and potentially of other porphyrins COPROI, UROI and UROIII [37].

While the last stages are still not fully resolved, recent studies substantiated the role of many actors in PpIX synthesis and iron metabolism as part of major protein complexes, which would most probably speed-up this last all-in-one stage completed by heme production. TMEM14C was described as essential to the synthesis of PpIX by facilitating the transport of protoporphyrinogen IX, but not of its precursors, into the mitochondria of erythroid cells, while no evidence was found in other cell types [38].

At this stage, the photoactive PpIX may be driven to the cytoplasm and outside of cells by an ABCG2 dimer localized in both mitochondrial and plasma membranes [39]. This multidrug efflux pump, while non-specific toward PpIX, is currently the most described way to regulate its level [40]. Recently, a study also described the involvement of exocytosis and showed that inhibition of dynamin 2, a major player in exocytosis, led to less extracellular and more intracellular PpIX levels [41].

Another transporter, ABCB1 (MDR1), is thought to transport PpIX through the mitochondrial and plasma membranes [42]. In this study, authors demonstrated that inhibition of the oncogenic Ras/MEK pathway restrained the efflux of PpIX due to ABCB1 level reduction in colon cancer cells. This was recently confirmed in a mouse malignant glioma where downregulation of MDR1 increased the cellular 5-ALA-induced PpIX level [43]. Researches on ABCB1 are very limited, hence its role remains to confirm.

It was recently found that ferrochelatase (FECH) homodimerizes to bind ABCB7 on one side and ABCB10 on the other side [44]. Furthermore, ABCB10 is known to bind and stabilize mitoferrin-1 (MFRN1, SLC25A37) an iron importer [45], suggesting a collaboration or close relationship between MFRN1-ABCB10 and ABCB10-FECH-FECH-ABCB7 to import iron and insert it in protoporphyrin IX through ferrochelatase. It is then hypothesized that ABCB7 enables ATP-driven ferrochelatase opening and subsequent iron protoporphyrin IX release [11,44].

ABCB8, another IMM actor, is becoming more and more attractive in heme, iron and cancer fields. It was recently proposed as a renal cell carcinoma prognostic marker. Knockdown of the corresponding gene reduced the migration and viability of renal cancer cell lines in vitro, suggesting a role in tumor progression [46]. It was also shown to mediate iron export in mitochondria of cardiomyocyte cells and to play an essential role in the maturation of iron-sulfur clusters (ISC) [47].

Neuropilin-1 (NRP1) is a transmembrane or cytosolic protein that acts on several signaling pathways, such as angiogenesis through VEGF and HGF in endothelial cells [48], and is a tumor promotor overexpressed in several tumor tissues [49,50,51]. Issit et al. revealed a physical interaction between NRP1 and ABCB8. In addition, their study indicated that NRP1 downregulation reduced ABCB8 expression and increased mitochondrial iron accumulation in endothelial cells. Analysis of iron transporters revealed that NRP1 knockdown also increased the level of the iron exporter FPN1 and the iron importer MFRN1. In contrast, TFR1 and MFRN2 iron importers were not deregulated [52].

**Figure 2:**
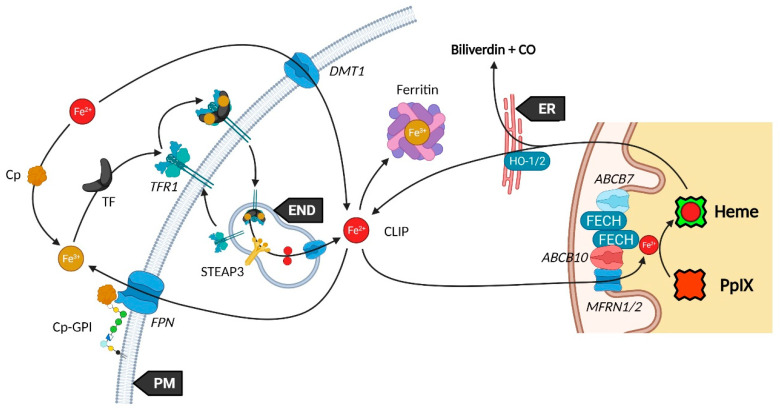
Utilization and regulation of the cytosolic labile iron pool.

Iron availability (see Figure 2) is tightly controlled by diverse mechanisms in cells that have been comprehensively reviewed [14,53,54]. Such control is mandatory to prevent Fe^2+^ accumulation that leads, through the Fenton reaction, to toxic hydroxyl radical production. Elementarily, ferrous iron (Fe^2+^) from the extracellular matrix binds to the transferrin (TF) carrier as ferric iron (Fe^3+^). Transferrin subsequently links to the plasma membrane transferrin receptor 1 (TFR1), forming a complex that will be endocytosed. Endosomal reduction of Fe^3+^ is handled by STEAP3, the six-transmembrane epithelial antigen of prostate 3 followed by translocation of free Fe^2+^ into the cytosol by the divalent metal transporter 1 (DMT1). DMT1 was also found to mediate Fe^2+^ intracellular level by acting as a plasma membrane importer. Similar to the Fe-TF-TFR1 complex, Fe-DMT1 can be internalized in endosomes and act on the cytosolic labile iron pool (CLIP) or deliver Fe to mitochondria. Additionally, DMT1 was found to be expressed in the outer mitochondrial membrane (OMM) and to act as both a mitochondrial iron importer and exporter [55]. A more direct use of the CLIP might take place throughout a “kiss and run” process that may operate by the sole touch of the endosome with the mitochondrion. The CLIP excess is controlled by the iron efflux protein ferroportin (FPN) that dispatches Fe^2+^ back in the cytoplasm, as well as by the iron storing complex ferritin [56]. Iron from the cytosolic pool may be incorporated in mitochondria through the MFRN1-ABCB10 complex, as mentioned above, in order to act as a substrate of PpIX.

Ceruloplasmin (Cp) is a potent copper sequestering protein that circulates in the blood flow. It shows a ferroxidase activity that is essential to the ferrous to ferric iron oxidation, a necessary step for transferrin (TFR1) loading and iron delivery inside cells [53,57]. Cp also mediates iron efflux by cooperating with ferroportin (FPN, IREG1) but must be glycosylphosphatidylinositol (GPI)-anchored to the plasma membrane next to FPN to activate the latter [58].

Heme subsequently moves to the cytoplasm to exert different functions. From this statement, it was proposed that heme cytoplasm homeostasis is controlled by mitochondrial exporters and cell membrane heme importers and exporters. The topic has been reviewed by Ponka et al. who blame a lack of evidence leading to several extrapolations [59].

Starting with mitochondrial export, FLVCR1b (Feline Leukemia Virus Subgroup C Cellular Receptors) is thought to make a way for heme to the cytoplasm considering that its expression is directly correlated to the cytoplasmic heme level [60]. However, no study corroborated this hypothesis and the localization on the mitochondrial membrane is not described yet. Additionally, it was recently noticed that FLVCR1b overexpression does not modify myoglobin heme-insertion [61]. Whether FLVCR1b directly transports heme is a mere assumption, but a secondary role for this protein is likely.

From the cytoplasm, heme might be either further expelled out to the extracellular matrix through FLVCR1a and ABCG2, or degraded into biliverdin and bilirubin while releasing its iron content and carbon monoxide (CO). The heme export role of FLVCR1a was proposed by Quigley et al. who showed that overexpression of FLVCR1a decreases the cytoplasmic heme level of rat epithelial cells incubated with the heme analog ZnMP [62]. Other studies reported a similar role, however, Destefanis et al. recently showed that FLVCR1a silencing did not lead to heme accumulation, both without and under 5-ALA treatment, in the SNU-407 colorectal adenocarcinoma cell line [63]. Nonetheless, a clear upregulation of the ABCG2 mRNA, a PpIX exporter, was found when FLVCR1a was silenced. Thus, it seems an equilibrium needs to be implemented as the lack of FLVCR1a could induce a porphyrin accumulation, even though this is not observed in the study.

FLVCR2 was suggested to import heme across the plasma membrane of mammalian cells [64]. Unlike its FLVCR1 homolog, an export role was not found for FLVCR2 (named FLVCRL14q) in vitro [62]. In contrast, no import feature was noticed in a Saccharomyces Cerevisiae yeast model [65].

Lately, Li et al. reported a new mode of action for FLVCR2 (named MFSD7C9) in an extensive study [66]. They first confirmed that FLVCR2 displays three binding sites to heme. Then, immunoprecipitation, subcellular fractionation coupled to Western blotting, FLVCR2-GFP and MitoTracker analysis confirmed a major localization to mitochondria, a transient interaction with energy transfer chain (ETC) complexes III, IV and V, and the endoplasmic reticulum ATPase SERCA2b implied in thermogenesis. Based on their results, they make the hypothesis that heme binding to FLVCR2 dissociates the latter from the ETC and SERCA2b that induces a mitochondrial respiration switch from ATP synthesis to thermogenesis.

In addition, they report an interaction between FLVCR2 and the heme catabolism proteins HO-1 and TFR1 [66]. Thus, FLVCR2 might be important for heme opening by HO-1 and Fe^2+^ homeostasis.

Heme-responsive gene 1 (HRG1) protein was shown to bind and transport heme while locating in endosomes and lysosomes [67]. In 2019, the same group demonstrated in a mice HRG1 KO model that heme accumulated in phagolysosomes of the reticuloendothelial system macrophages [68]. An over 10-fold accumulation in heme was observed, and the size of the lysosomes was enlarged from 10 to 100-fold compared to normal mice. This storage was explained by a crystallization of heme into hemozoin, potentially to avoid heme toxicity [68].

In 2013, a team related the HRG1 plasma membrane localization with a high degree of cell invasive and migratory features. They hypothesize that HRG1 induces the vacuolar-(H^+^) ATPase that co-expresses to the plasma membrane, whose role in pH regulation, glucose metabolism and metalloproteinases activities may regulate the metastatic ability of cancer cells [69]. Altogether, this suggests a direct role of heme metabolism regulation into cancer cells behavior.

Heme carrier protein 1 (HCP1), a transmembrane proton-coupled folate transporter (PCFT) was found to be a potent intestinal heme transporter [70]. More recently, an in vivo study displayed a role in hepatocyte iron regulation [71]. HCP1 gene (SLC46A1) silencing was associated with a reduced liver iron level and increased TFR1 and FPN protein expressions. They confirmed that HCP1 in hepatocytes was able to transport heme, however heme treatment decreased HCP1 expression in hepatic cell line [71].

Progesterone receptor membrane component 1 (PGRMC1) is another regulator of the heme metabolism. Its role in heme regulation through FECH interaction was described in human cells by Piel et al. who also suggested a direct interaction between PGRMC1 and heme [72]. More recently, human PGRMC1 was confirmed to link to both ferrous and ferric forms of heme [73] and to bind to various cytochromes P450 in a heme-independent fashion [74].

Some players and their attributed role can now be trusted; however, many others are either disputed or not sufficiently examined. The overall comprehension of the transport mechanisms of heme-related actors is still faraway and extrapolations should be handled with great care.

## 3. Concerning PpIX Selective Accumulation in Neoplastic Cells

### 3.1. Enzymes: The Prime Targets

Enzymes regulating the heme biosynthesis have been studied for long time, as they are assumed to give rise to PpIX accretion mechanism observed in cancer cells following exogenous administration of 5-ALA.

The simple assumption that a particular enzyme be up or down regulated in neoplasms derives from the actual observation of such modifications in porphyria. Even though studies have been very consistent on which disease involves which enzyme, it is disappointing to notice that this rule does not apply to cancer and that not much elements have been widely agreed. Reasons for such discrepancies are manifold: unlike models, measurement methods and cell levels, uneven doses and time measurement, and diverse inhibitors.

**Figure 3:**
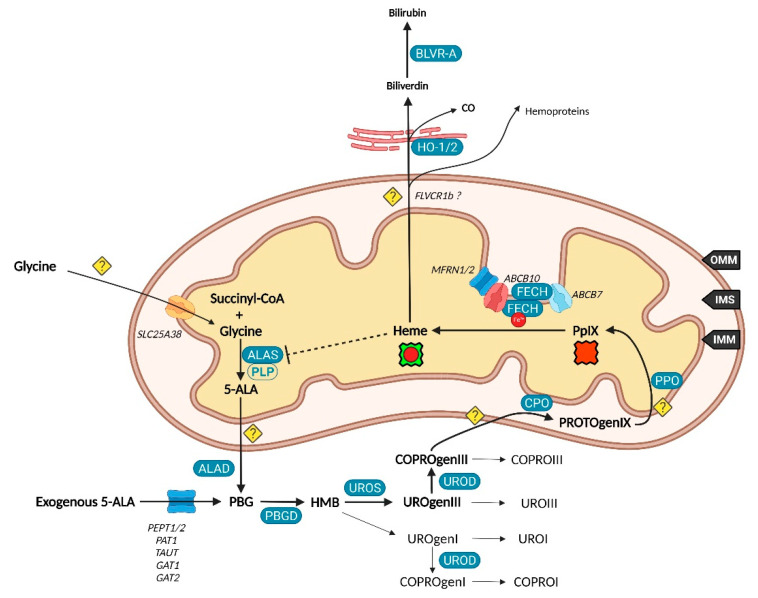
Ten companions, the fellowship of the heme.

The very first enzyme of the heme cycle ALAS and its cofactor pyridoxal-5′-phosphate (PLP) are mandatory to 5-ALA synthesis (see Figure 3). Indeed, heme synthesis must take place in most eukaryote cells mainly to provide respiratory cytochromes. ALAS, that is for long considered the rate-limiting catalyzer on the heme route, is characterized in mammals by the two isoforms ALAS1 and ALAS2. The first one is encoded on the chromosome 3p21 and ubiquitous in all cells, while ALAS2 on the chromosome X is solely expressed in erythroid cells [75]. ALAS is besides the only enzyme of the cycle whose defect does not lead to porphyria. Furthermore, its inhibition prevents heme synthesis as performed by the latter to self-regulate, making it a prime choice in defective heme biosynthesis studies. ALAS1 regulation can take place in various manners such as transcriptional repression [76]. As a matter of fact, ALAS1 import to the mitochondria is inhibited by heme binding through its heme regulatory motifs (HRM), while HRM mutations allowed ALAS1 mitochondrial accumulation [77].

One could assume that overexpression of ALAS could counteract inhibition and induce the accumulation of heme and of its fluorescent precursor PpIX. However, both ALAS overexpression and 5-ALA exogenous administration failed to enhance PpIX level in healthy cells.

Recently, non–small cell lung cancer (NSCLC) cell lines stably overexpressing ALAS1 by lentiviral vector transfection displayed enhanced tumorigenesis characterized by heme overexpression, increased oxygen consumption and ATP production, higher migration and invasion, as well as bigger tumor size in mice [78].

ALAS2 is essential to erythropoiesis and its regulation appears to be dependent on multiple factors at the transcriptional, translational, and post-translational levels. Recently, a study unveiled that UCA1 (Urothelial cancer-associated 1) gene, a long-non-coding RNA, stabilizes ALAS2, thus enabling heme biosynthesis [79]. The regulation of ALAS was fully reviewed by Peoc’h et al. lately [80].

ALA dehydrase (ALAD) is a zinc-dependent enzyme switched on by Zn^2+^ and Al^3+^ while lead Pb^2+^ and Succinyl acetone (4,6-dioxoheptanoic acid) act as inhibitors and prevent porphobilinogen synthesis. Little is known about the role of ALAD, also called PBGS, in cancer. A recent study found that ALAD expression was lowered in breast cancer cells compared to normal breast cells. Furthermore, ALAD seems to inhibit the transforming growth factor-b (TGF-b), a tumor suppressor involved in epithelial to mesenchymal transition (EMT) [81]. Interestingly, it was reported that ALAD is straight inhibited by protoporphyrin IX in Escherichia coli [82]. These findings could suggest a direct modulation of tumor growth through ALAD expression, the first enzyme involved in the heme synthesis pathway bypassed by ALA exogenous administration. It should be noted that ALAD activity has been investigated using tryptophan, its inducer, but that the tryptophan interactions with other enzymes are not entirely appreciated [83].

In spite of an obvious communication between the heme biosynthesis and the iron-sulfur biogenesis, it is only in 2020 that Liu et al. demonstrated a direct relation between both systems [84]. They actually found that the Fe_4_S_4_ cofactor is required for an optimal ALAD activity. The involvement of oxidative stress was also raised as both Fe-S clusters and ALAD are sensitive to the latter. 

The use of 5-ALA for breast cancer PDD had already been proposed by Millon et al. who found that, despite the production of PpIX not only happened in breast cancer but also in normal breast cells, the significant relative change in fluorescence allowed discrimination between both types. Thus, 5-ALA-mediated fluorescence is presented as a potential tool for breast cancer diagnosis [85].

Another in vitro analysis clarified the role of ALAD, PBGD and FECH in breast cancer by silencing their expression [86]. As in the study described above, ALAD silencing greatly reduced PpIX level after 5-ALA treatment. The same trend was observed with PBGD. However, Yang et al. did not monitor any PpIX level change in the untreated cells for these treatments. This suggests that the heme production usually works at reduced speed at which ALAD and PBGD are not saturated, while their saturation by 5-ALA administration may be responsible for the reach of a PpIX limit level. While FECH silencing allowed a significant PpIX accumulation in untreated cells, it was barely increased after 5-ALA treatment. In a more recent study, the same group showed that chelation of iron by deferoxamine increased fluorescence intensity in various breast cancer cell lines such as SKBR3 and in the non-cancerous line MCF10A. FECH-knockdown in SKBR3 also increased PpIX levels; however, deferoxamine treatment of these modified cells did not induce a PpIX change [87]. Thus, one can assume that PpIX accumulation used in PDT and PDD is mainly due to a change in FECH amount or activity. 

A major point that should be highlighted when trying to compare inter-studies results is the growth state of cell models. Krieg et al. [88] investigated this parameter in colon carcinoma cells and found many disparities. While a difference in 5-ALA uptake from one cell line to another seems obvious, they found that this uptake greatly varies in some cells, depending on their growth state, i.e., either in exponential or plateau phase. Surprisingly, PpIX formation, while significantly higher in all cancer cells, was not necessarily correlated to 5-ALA uptake efficacy. Study of PBGD showed a higher activity in all colon cancerous cells compared to normal colon cells, irrespective of their growth state. However, the normal cell line had the highest relative FECH level. They concluded that the selective accumulation of PpIX is not the result of PBGD and FECH regulation, but is part of a larger and more complex process.

Unfortunately, enzymes UROS and UROD have not been intensively studied in mammals although their role in heme cycle appears not to be trivial. UROS deficiency does not allow further uroporphyrinogen III synthesis and leads to a pre-uroporphyrinogen accumulation able to form uroporphyrinogen I chemically. Nonetheless, this isomer is not involved in the formation of UROIII [89] and ends with the uroporphyrin I and coproporphyrin I by-products. In spite of the lack of interest in these marginal branches, it was recently shown that the 610–650 nm range fluorescence emission (405 nm excitation) was significantly higher in the plasma of colorectal adenocarcinoma patient than in control group. Furthermore, studying porphyrin content unveiled that this fluorescence was due to the protoporphyrin IX as well as the coproporphyrin I [90]. It had already been found that blood porphyrin fluorescence is a breast cancer marker [91] and that coproporphyrin and PpIX accumulate specifically in oral squamous cell carcinoma [92].

It can be suspected that the accumulation of 5-ALA-induced heme precursors is partly regulated through the conversion of hydroxymethylbilane to the fluorescent end-product coproporphyrin I.

5-ALA was also shown to be a potent tool for bladder cancer diagnosis. Inoue et al. [93] analyzed urine from bladder cancer patients to compare porphyrin levels. They found that total porphyrin amount excreted was significantly higher in cancer patients compared to healthy ones, both at 4 h and 8 h post treatment. That was mainly represented at 8h by UROI and COPROI, two porphyrins formed upstream of the UROS stage with high sensitivity and specificity, while UROIII and COPROIII produced downstream were not dysregulated. This suggests that UROS activity was rate-limiting in bladder cancer patients and that the accumulation of hydroxymethylbilane is regulated through its spontaneous cyclization into UROgenI.

Whether UROD activity is altered in tumors is not clear yet due to a lack of consideration. A UROD^+/−^ murine model of porphyria cutanea tarda (PCT), a genetic disease caused by UROD loss of activity, induced a 2-fold UROD activity decrease, which did not result in hepatic porphyrins accumulation, indicating the enzyme is not rate limiting [94]. Later, the same group found that uroporphomethenes, inhibitors of UROD, were produced in hepatocytes by oxidation of uroporphyrinogens in an iron-dependent manner [95]. However, since the discovery of this type of inhibitors, no additional report has been made on it.

The next enzyme in the cycle, CPO, is located in the mitochondrial intermembrane space [96], anchored to the IMM [97] and is thought to play a major role in heme level regulation. In 1998, Ortel et al. showed that calcium-dependent differentiation of normal keratinocyte induced a strong PpIX expansion, associated with an increase in CPO mRNA [98]. Módis et al. recently demonstrated the involvement of Cystathionine-γ-lyase (CSE), an important hydrogen sulfide (H2S) producer in mitochondrial regulation [99]. They found that CSE silencing enhanced mitochondrial function by increasing plasma and liver heme levels in mice. In addition, they correlated the lowered CSE level with a CPO overexpression in the liver of CSE-/- mice (ALAD and UROD were not modulated but FECH was increased although not significantly). It was supported by the in vitro analysis of HepG2 liver cells. Indeed, an H2S-inducer lowered CPO promoter activity while a CSE inhibitor increased it. They suggested a relationship between the CSE and CPO systems on the mitochondrial bioenergetics aspect. Other roles have been proposed for H2S such as hemoprotein ligand associated with a RedOx process [100] as well as improvement of heme degradation. The latter could occur through an O_2_-independent mechanism thus relieving HO-1 activation [101] and the HO-1 upregulation [102]. To our knowledge, it seems that this point has not been investigated in the context of 5-ALA-PDD or PDT yet, although it could be a key clue of the PpIX selectivity.

The penultimate stage involves PPO, that was thought to be attached to the IMM and localized in the IMS. However, it was recently found that PPO resides in the mitochondrial matrix [97], implying that PROTOgenIX and not PpIX must move across the IMM. PPO is known to be overexpressed during erythroid differentiation while CPO is downregulated [103]. It was recently suggested that CPO and PPO are differentiation markers. In early gastric cancer, both of them were found overexpressed in highly differentiated types compared to poorly differentiated ones [104]. A noteworthy mechanism is responsible for the usability of PPO inhibitors as sole photodynamic agents. While PpIX synthesis is blocked, PROTOgenIX accumulates in the mitochondrial intermembrane space and spreads in the cytoplasm where it undergoes oxidation leading to PpIX accumulation. In addition, this PpIX is not a substrate of mitochondrial FECH anymore and thus accumulates although no negative feedback is exerted on ALAS [105].

PPO expression was found to be higher in rapidly growing colorectal cell lines and its inhibition significantly reduced the tumor growth rates in vivo [106]. CPO and PPO expressions can also be influenced by iron concentration. Indeed, its deficiency in rat livers leads to the overexpression of both CPO and PPO while iron overloading decreases CPO, FECH and the heme content but not PPO [107].

FECH is finally mandatory to mediate PpIX to heme conversion by inserting ferrous iron in the porphyrin cycle. This action was suggested to be supported by frataxin whose iron binding ability could provide FECH with the necessary Fe^2+^ [108]. In 2013, Sawamoto et al. found that its upregulation reduced the 5-ALA-induced accumulation of PpIX while its downregulation was related to PpIX accumulation [109]. However, no study reinforced this mechanism recently, while most of them claim a role in iron-sulfur cluster synthesis [110].

Besides ALAD, FECH is also a Fe-S dependent enzyme [111]. Deregulation of the heme biosynthesis observed in neoplasms may occur through the ISC biogenesis modulation and direct FECH expression drop.

Another mode of action for FECH was raised by Taketani et al., who demonstrated a reverse activity, that is iron removal from the heme [112]. This group also showed an additional localization of FECH to the outer mitochondrial membrane of mammalian cells and a potential effect of FECH phosphorylation on its location and activity [113]. In addition, they reported fatty acids and phospholipids to enhance FECH activity in porcine tissues [114]. We are concerned not to see other studies corroborating this reverse activity, and call for deeper studies using alternative techniques. In fact, the role of lipids in FECH activity of rat liver had already been described [115].

While many laboratories endeavor to unearth enzyme level variations in the heme metabolism, one must note that the enzyme activity regulation may be of higher importance.

HO-1 is an established key element of the heme degradation, making relevant the reasoning that it is downregulated in cancer cells, inducing heme and ultimately PpIX accumulation upon 5-ALA treatment. Actually, HO-1 was reported to be upregulated in various cancer cells [116]. This observation addresses an issue as it suggests that the heme catabolism is enhanced in cancer cells and therefore the PpIX accumulation reduced. 

Approaching the question from another angle, HO-1 was found to be involved in Fe-ATPase operation, an iron exporting pump [117,118]. In parallel, there is a recurring observation that cancer cells display high levels of iron. The HO-1 overexpression might be directly involved in the iron elimination strategy to prevent toxicity.

### 3.2. Transporters: Why No Greater Interest?

ABCG2 is known for its role in PpIX export [119] but whether it is involved in 5-ALA mediated PpIX selective accumulation in cancer cells is unclear. Kobuchi et al. already investigated this topic on different cancer cells and found an important variation in ABCG2 expression from one cell type to another [39]. More recently, Morita et al. investigated the selective accumulation of PpIX in breast cancer cells from a tumor-bearing mouse model [120]. In a confocal fluorescence microscopy assay, they found that MCF7 and MDA-MB-231 did highly respond to 5-ALA stimulation 2 h after treatment unlike the normal epithelial MCF10A cells, supporting a selectivity for breast cancer. However, this observation only reflects the PpIX amount at a certain time point as it might take longer to MCF10A cells to accumulate PpIX. The size of the cells should also be considered as for an equal PpIX amount, the bigger the cell the lower the mean fluorescence. In contrast, cytometry investigations revealed a significant response from MCF10A to 5-ALA stimulus. Subsequently, they looked at the role of ABCG2 thanks to its specific inhibitor Ko143. They found that the combination of 5-ALA along with Ko143 increased PpIX level even more while lessening the inter-cellular fluorescence variability. This last feature could be due to the reach of a plateau in the PpIX response to the dose dependent Ko143 stimulation, indicating a saturation of the heme production rate. It is not clear whether there is a significant change from 5-ALA to 5-ALA + Ko143 treatment and thus if ABCG2 inhibition is truly relevant for selective PpIX accumulation. In fact, considering ABCG2 exports PpIX, the level of this transporter should be higher in normal cells than cancer cells in order to observe a higher fluorescence rise in the latter. Thus, if a role for ABCG2 in PpIX mediation seems clear, its expression level in the different cell lines do not explain PpIX selective accumulation in cancer cells after 5-ALA treatment. 

NRP1 is a tumor promotor overexpressed in several tumor tissues such as oral squamous cell carcinoma [49], breast [50] and lung [51] cancers. Interestingly, only one study was found to connect NRP1 to heme synthesis. Sollwedel et al. showed that HO-1 induction by Cobalt-Protoporphyrin increases NRP1 mRNA level in a pregnant mice model [121]. Thus, 5-ALA-induced HO-1 overproduction may increase NRP1 and subsequently ABCB8 levels as described above, leading to reduced mitochondrial iron content and PpIX accumulation.

Ogura has been exploring the role of transporters involved in the heme metabolism very attentively [122]. Lately, his group demonstrated the importance of PAT1 and PEPT1 upon 5-ALA administration, depending on the malignancy of the DU145 prostate cancer cell line [123]. PAT1 inhibition by tryptophan induced lower PpIX accumulation at 24 h only in high malignancy condition. Supposedly, PAT1 may not be engaged in 5-ALA import in non-malignant cells, thus limiting their PpIX anabolism. However, PEPT1 inhibition by ibuprofen showed a hindered PpIX level in both malignancy levels, even though the lowest seemed faintly affected. This suggests that PEPT1 may also serve as a gatekeeper whose differential expression could be a reason for the high selectivity of neoplasms toward healthy cells. Attention should be paid when using tryptophan as its range is not comprehensively known. For instance, it was recently mentioned that tryptophan inhibits ALAS and potentially further enzymes of the cycle [83].

The involvement of PEPT1 had already been raised in other prostate cancer cells (PC-3) [124] as well as in gastric cancer cells [125].

TMEM14C is an IMM protein that was reported to facilitate the import of PROTOgenIX into the mitochondrial matrix of erythroid cells [38]. Indeed, mice fetal liver tissue and erythroid cells deficient in TMEM14C had their PpIX synthesis reduced and accumulated COPROIII, suggesting the accretion of the upstream intermediate COPROgenIII and a potential PROTOgenIX production hindrance.

TMEM14C was also found to be a head and neck squamous cell carcinoma prognostic marker when overexpressed [126]. We can assume that this upregulation in cancerous cells is related to an enhanced PpIX anabolism and could be a reason for the selective accumulation of PpIX post-5-ALA treatment.

## 4. Conclusions and Perspectives

The improvement and emergence of innovative technologies allowed researchers to delve the heme biosynthesis pathway more and more in recent years, providing new data, uncovering previously unknown transporters and metabolites. To date though, we feel that knowledge about paths that surround the heme metabolism has solely thinly progressed. For if a tremendous information has been gained, discrepancies amongst studies are now commonplace. They may originate from the comparison of dissimilar models, to the generalization of results obtained in a particular case, or to the progressive acceptance of hypothesis that have not been confirmed by additional peer studies.

In this review, we first tried to gather the accepted actors surrounding the heme and to display their interactions in a sole fashion. Subsequently, we reconsidered the possible explanations for the selectivity of 5-ALA-induced PpIX accumulation towards cancers.

The direct production of heme is rather well-known, from the use of the glycine substrate, eight enzymes are involved. 5-ALA exogenously administered circumvents the negative feedback exerted by heme on its own production by inhibition of the first enzyme. The hydroxymethylbilane intermediary may escape the main path and ultimately generate two porphyrins, UROI and COPROI, by spontaneous cyclization. How the successive molecules span the mitochondrial membranes, either with the export or import intent, is nevertheless ambiguous. This is a crucial point to understand the limiting steps and try to improve the selective accumulation of protoporphyrin IX in neoplasms. It is possible that previously described membrane proteins are facilitators of mitochondrial penetration and that changes of lipophilicity along reactions, in relation with the changing of pH between cellular compartments, is responsible for the penetration enhancement of the target intermediary. Despite our model displays CPO, PPO and FECH away from each other, it is likely that they locate in their very vicinity to provide a quick conversion of COPROgenIII into PpIX. How the heme pool balance is controlled is slightly blurry as well. It can move out to the cytoplasm and release its iron core that subsequently reconstitutes the iron pool, while turning into biliverdin and bilirubin. What happens with bilirubin in cells, besides hepatocytes, is unknown. A further degradation into smaller metabolites is plausible. 

Finally, the question of the selective accumulation of PpIX in neoplasms is still pending. Some transporters and enzymes demonstrate relevant behaviors. However, other studies invalidate the idea of their central role in selectivity regulation. In order to solve the biggest question of the PDT history, we urge our peers to engage in wider studies using not only one or two cellular models but several that will enable safer extrapolations and render inter-studies comparisons easier.

## Abbreviations

**Metabolites**	
5-ALA	5-aminolevulinic acid/aminolevulinate
COPRO	coproporphyrin
URO	uroporphyrin
PBG	porphobilinogen
HMB	1-hydroxymethylbilane/pre-uroporphyrinogen
UROgen	uroporphyrinogen
COPROgen	coproporphyrinogen
PROTOgen	protoporphyrinogen
PpIX	protoporphyrin IX
**Organelles**	
PM	plasma membrane
ER	endoplasmic reticulum
GA	Golgi apparatus
OMM/IMM	outer/inner mitochondrial membrane
IMS	inner mitochondrial space
END	endosome
LYSO	lysosome
**Enzymes**	
ALAS	ALA-synthase
PLP	pyridoxal-5’-phosphate
ALAD	ALA-dehydratase
PBGD	porphobilinogen deaminase
UROS	uroporphyrinogen III cosynthase
UROD	uroporphyrinogen decarboxylase
CPO	coproporphyrinogen III oxidase
PPO	protoporphyrinogen oxidase
FECH	ferrochelatase
HO	heme oxygenase
BVRA	biliverdin reductase-A
UGT1A1	UDP-glucuronosyltransferase 1A1
**Others**	
PDT	photodynamic therapy
PDD	photodynamic diagnosis
ROS	reactive oxygen species
TCA	tricarboxylic acid
CLIP	cytosolic labile iron pool
ISC	iron-sulfur cluster
CO	carbon monoxide
ABCB	ATP-binding cassette subfamily B member
